# Fyn Kinase regulates GluN2B subunit-dominant NMDA receptors in human induced pluripotent stem cell-derived neurons

**DOI:** 10.1038/srep23837

**Published:** 2016-04-04

**Authors:** Wen-Bo Zhang, P. Joel Ross, YuShan Tu, Yongqian Wang, Simon Beggs, Ameet S. Sengar, James Ellis, Michael W. Salter

**Affiliations:** 1 Program in Neurosciences & Mental Health, The Hospital for Sick Children, Toronto, ON, M5G 0A4, Canada; 2 Department of Physiology, University of Toronto, Toronto, ON, Canada; 3 Program in Developmental & Stem Cell Biology, The Hospital for Sick Children, Toronto, ON, M5G 0A4, Canada; 4 Department of Molecular Genetics, University of Toronto, Toronto, ON, Canada

## Abstract

NMDA receptor (NMDAR)-mediated fast excitatory neurotransmission is implicated in a broad range of physiological and pathological processes in the mammalian central nervous system. The function and regulation of NMDARs have been extensively studied in neurons from rodents and other non-human species, and in recombinant expression systems. Here, we investigated human NMDARs *in situ* by using neurons produced by directed differentiation of human induced pluripotent stem cells (iPSCs). The resultant cells showed electrophysiological characteristics demonstrating that they are *bona fide* neurons. In particular, human iPSC-derived neurons expressed functional ligand-gated ion channels, including NMDARs, AMPA receptors, GABA_A_ receptors, as well as glycine receptors. Pharmacological and electrophysiological properties of NMDAR-mediated currents indicated that these were dominated by receptors containing GluN2B subunits. The NMDAR currents were suppressed by genistein, a broad-spectrum tyrosine kinase inhibitor. The NMDAR currents were also inhibited by a Fyn-interfering peptide, Fyn(39–57), but not a Src-interfering peptide, Src(40–58). Together, these findings are the first evidence that tyrosine phosphorylation regulates the function of NMDARs in human iPSC-derived neurons. Our findings provide a basis for utilizing human iPSC-derived neurons in screening for drugs targeting NMDARs in neurological disorders.

NMDA receptors (NMDARs) are a prominent subtype of ionotropic glutamate receptor mediating fast excitatory synaptic transmission in the central nervous system (CNS)^1^. NMDARs are cation channels, permeable to Ca^2+^, Na^+^, and K^+^, and play key roles in synaptic plasticity^2^, physiological and pathological events that modify the strength of synaptic transmission. The functional receptors are heterotetramers composed of two necessary GluN1 subunits and two GluN2 subunits^2^. NMDARs containing the GluN3 subunit have also been described^2^. GluN1 subunits, which contain co-agonist glycine binding sites, have eight distinct splice variants and are encoded by a single gene. GluN2 subunits, at which glutamate binding sites are located, contain four subtypes (GluN2A-D) and are encoded by four different genes. The GluN2 subunits have a key role in determining NMDAR properties, such as receptor gating, pharmacology, Ca^2+^ permeability, and blockade by extracellular Mg^2+^, leading to heterogeneity in NMDAR function across the CNS.

NMDARs are broadly expressed in various types of neurons, and their function and regulation have been extensively investigated in neurons from rodents and other non-human species^2^,^3^. Also, human NMDAR properties have been studied in recombinant expression systems^4^. From studies using non-human neurons, it is well-known that the function of NMDARs is regulated by members of the Src family of non-receptor protein tyrosine kinases^5^,^6^. Src family kinases contribute to physiological and pathological changes in synaptic strength by modulating NMDAR function and thus are involved in brain development, learning and memory formation, and pathogenesis of neurological disorders, such as chronic pain and schizophrenia^7^,^8^. Src family kinase regulation of NMDARs may therefore be a target for potential therapeutics in the treatment of neurological disorders^1^.

Induced pluripotent stem cells (iPSCs), produced by reprogramming adult fibroblasts^9^, have pluripotency and can be differentiated into any type of cell including functional neurons. Studies from various groups^10^,^11^,^12^,^13^,^14^ have provided a strong basis for utilizing iPSC-derived neurons to study neurological diseases for exploring pathophysiological mechanisms. Previous studies have shown that human iPSC-derived neurons can be used for functional studies of NMDARs^15^,^16^, but the regulation of NMDARs in these neurons is unexplored. In the present study we investigated the function and regulation of human NMDARs using human iPSC-derived neurons. We found that GluN2B subunit-containing NMDARs were dominant and that tyrosine-protein kinase Fyn potentiated the function of GluN2B subunit-containing NMDARs in human iPSC-derived neurons.

## Results

### Characteristics of human induced pluripotent stem cell-derived neurons

As a first step in characterizing the function of iPSC-derived neurons, we generated iPSCs from a healthy adult female with no known neurological or psychiatric disorders. Dermal fibroblasts were reprogrammed using retrovirus-mediated delivery of *OCT4*, *SOX2*, *KLF4*, and *MYC* (OSKM)^17^, resulting in three iPSC lines. These iPSCs repressed the viral reprogramming factors (Supplementary Fig. S1), activated the endogenous OSKM genes, synthesized the pluripotency-associated proteins NANOG, TRA1-60, SSEA4, and OCT4, and also had normal karyotypes (Supplementary Fig. S1). Pluripotency was functionally demonstrated both *in vitro* and *in vivo*, by the ability of these iPSCs to commit to the three germ layers in embryoid body and teratoma assays, respectively (Supplementary Fig. S1). Together, these data confirm that selected iPSC lines are pluripotent.

**Figure 1:**
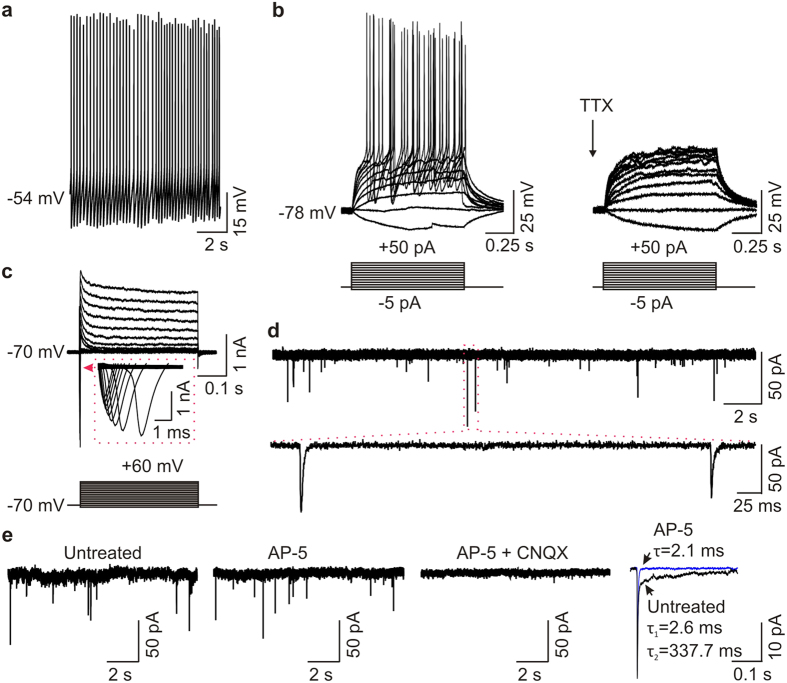
Electrophysiological characteristics of human iPSC-derived neurons. (**a**) Representative traces showing that a human iPSC-derived neuron displays spontaneous action potentials. (**b**) Typical traces display action potentials evoked by injecting a series of current steps from −5 pA to +50 pA for 1 s in a human iPSC-derived neuron and the action potentials were eliminated by TTX (0.5 μM). (**c**) Representative traces showing voltage-gated Na^+^ currents and K^+^ currents elicited by stepping the membrane potentials to a series of potentials from −80 mV to +60 mV for 400 ms in a human iPSC-derived neuron. (**d**) Spontaneous synaptic activities in the presence of TTX (0.5 μM) in a human iPSC-derived neuron. (**e**) Representative traces showing mEPSCs under the conditions of untreated (left), treated by AP-5 (100 μM, middle), and treated by AP-5 + CNQX (10 μM, right) in a human iPSC-derived neuron. Inset (far right): averaged mEPSCs before (untreated, black) and during (blue) the application of AP-5 in this cell.

We next directed neuronal differentiation in three iPSC lines using our previously published protocol^11^, which yields a mixture of cells with gene expression characteristics of neurons in the upper (~30%) and lower (~60%) layers of the cortex^11^. Using this protocol, these neurons are a mixture of glutamatergic (~35%) and GABAergic (~45%) neurons^11^. Immunostaining studies showed that these iPSC-derived cells had neuronal morphology and expressed the neuron-specific protein MAP2 (Supplementary Fig. S2). For electrophysiological recordings, we chose just one to a few of the neurons with the most typical neuronal morphology in each dish. With morphological identification, action potentials could be evoked in each of the 10 cells tested (*n* = 10; Fig. 1a,b and Supplementary Table S1). Injection of current steps evoked a single or train of action potentials in each of the 10 cells tested. Of the 5 cells that responded with a train of action potentials, three of the cells displayed spike frequency adaptation (Supplementary Fig. S3). The action potentials were abolished by tetrodotoxin (TTX, 0.5 μM), a blocker of selective, fast Na^+^ channels (Fig. 1b). Consistently, human iPSC-derived cells expressed voltage-gated Na^+^ currents and voltage-gated K^+^ currents (Fig. 1c). In addition, using whole-cell voltage-clamp recordings we found that the cells had spontaneously occurring miniature excitatory postsynaptic currents (mEPSCs) (Fig. 1d,e) indicating that the cells had functional synapses. The mEPSCs displayed two components: a fast component blocked by CNQX (10 μM), a potent antagonist of AMPA/kainate glutamate receptors, and a slow component blocked by AP-5 (100 μM), a competitive antagonist of NMDARs (Fig. 1e). These findings demonstrate that human iPSC-derived cells are functional neurons.

**Figure 2:**
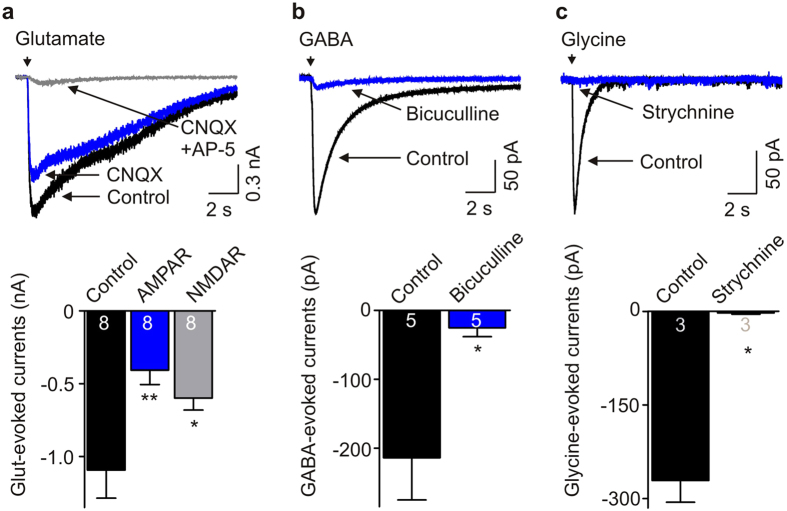
Human iPSC-derived neurons express functional ligand gated ion channels. (**a**) Representative traces and histograms showing glutamate-evoked currents in human iPSC-derived neurons and the effects of CNQX and AP-5 on the currents. AMPAR: CNQX-sensitive currents, NMDAR: AP-5-sensitive currents. *n* = 8, *P* = 0.005 using one-way ANOVA (*P < 0.05 and **P < 0.01, compared with control). (**b**) GABA-evoked currents in human iPSC-derived neurons and the currents were blocked by bicuculline at 10 μM. *n* = 5, *P = 0.02. (**c**) Typical traces and histogram showing glycine-evoked currents in human iPSC-derived neurons and the effects of strychnine at 1 μM on the currents. *n* = 3, *P = 0.02.

We then examined ligand-gated ion channels in these human iPSC-derived neurons by making whole-cell patch-clamp recordings. We found that applying glutamate induced currents in 8 of 9 cells tested (Fig. 2a; mean: −1.09 ± 0.19 nA). Glutamate-evoked currents were decreased by the application of the AMPA receptor antagonist CNQX (10 μM): mean AMPAR-mediated current was −0.41 ± 0.10 nA. The remainder of the currents were blocked by the NMDAR antagonist AP-5 (100 μM): mean NMDAR-mediated current was −0.60 ± 0.08 nA. We also found that application of GABA, in the presence of SCH 50911 (10 μM; a selective antagonist of GABA_B_ receptor), induced currents in 5 of 6 neurons tested (Fig. 2b). The current-voltage relationship showed that the GABA-evoked currents had a reversal potential at approximately −59 mV (Supplementary Fig. S4). Moreover, GABA-induced currents were inhibited by 10 μM bicuculline (−214.0 ± 61.1 pA, control *versus* −25.2 ± 12.8 pA, with bicuculline; *n* = 5, *P* = 0.02), a selective antagonist of GABA_A_ receptors (Fig. 2b). In addition, applying glycine induced strychnine-sensitive currents (−271.0 ± 34.9 pA, control *versus* −2.3 ± 2.3 pA, with strychnine; *n* = 3, *P* = 0.02) in 3 of 3 human iPSC-derived neurons tested (Fig. 2c), which had linear current-voltage relationship and reversed at about −55 mV (Supplementary Fig. S4). These data indicate that human iPSC-derived neurons express functional GABA_A_ and glycine receptors, in addition to AMPA and NMDA receptors.

### GluN2B subunit-containing NMDARs dominate in human iPSC-derived neurons

**Figure 3:**
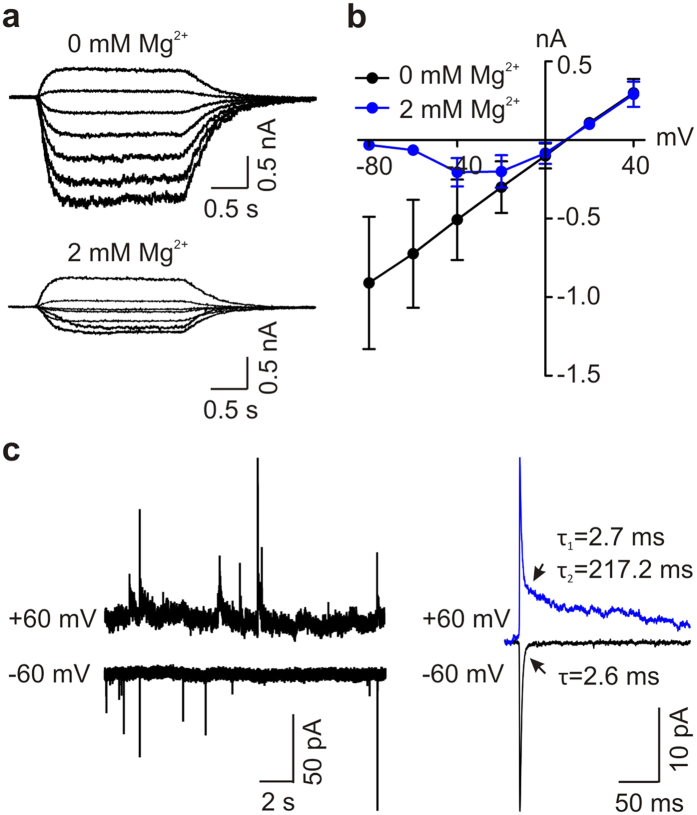
Extracellular Mg^2+^ causes voltage-dependent block on NMDA-evoked currents and synaptic NMDAR currents in human iPSC-derived neurons. (**a**) Representative traces showing agonist-evoked NMDAR currents elicited at a series of membrane potentials from −80 mV to +40 mV (in 20 mV increments) in the absence (upper) and presence (bottom) of 2 mM Mg^2+^ in a human iPSC-derived neuron. (**b**) A plot showing current-voltage relationship of NMDAR currents in the absence and presence of 2 mM Mg^2+^ in human iPSC-derived neurons. *n* = 3. (**c**) Left, Typical trace showing mEPSCs recorded at the membrane potentials of −60 mV (bottom) and +60 mV (upper) in presence of extracellular Mg^2+^ at 2 mM in a human iPSC-derived neuron. Right, Averaged mEPSCs in this cell at the membrane potentials of −60 mV and +60 mV.

Next, we examined in more detail the properties of the NMDARs in human iPSC-derived neurons. One striking feature of NMDAR-mediated currents is that the currents have voltage-dependent block by Mg^2+^ in the extracellular solutions^18^. We found that the current-voltage relationship for NMDA-evoked whole-cell currents was linear in human iPSC-derived neurons in the absence of extracellular Mg^2+^ and that extracellular Mg^2+^ (2 mM) caused a voltage-dependent block of the currents (Fig. 3a,b). Consistently, extracellular Mg^2+^ also caused a voltage-dependent block of the NMDAR-mediated slow component of mEPSCs (Fig. 3c).

**Figure 4:**
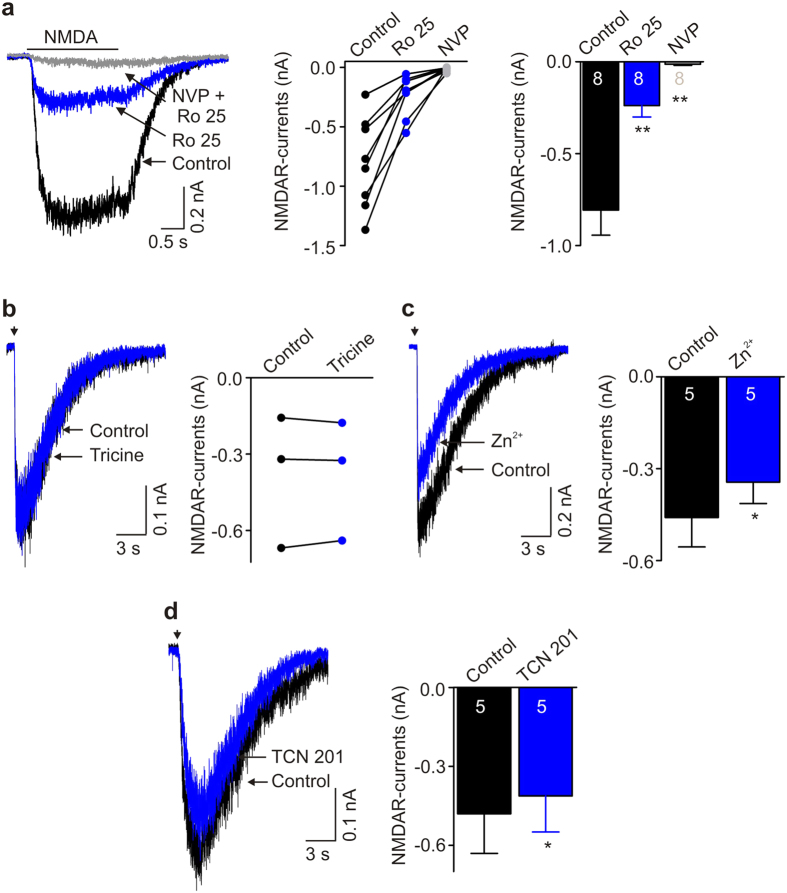
GluN2B subunit-containing NMDARs are dominant in human iPSC-derived neurons. (**a**) Left, Traces showing agonist (50 μM NMDA)-evoked NMDAR currents under control, treated by Ro 25-6981, and treated by NVP-AAM077 + Ro 25-6981 in a human iPSC-derived neuron. Middle, A graph shows NMDAR currents before and after adding Ro 25-6981 and NVP-AAM077 + Ro 25-6981 in 8 recorded human iPSC-derived neurons. Right, Histogram shows NMDAR currents under control, treated with Ro 25-6981 and treated with NVP-AAM077 + Ro 25-6981. *n* = 8, **P < 0.01, compared with control. (**b**) Representative traces (left) and graph (right) showing NMDAR currents before and after adding tricine (10 mM). *n* = 3, *P* = 0.9 using paired *t-*test. (**c**) Typical traces (left) showing NMDAR currents before and after adding 200 nM free Zn^2+^. Histogram (right) shows the effect of Zn^2+^ on NMDAR currents in human iPSC-derived neurons. *n* = 5, *P = 0.014 using paired *t-*test. (**d**) Typical traces (left) showing NMDAR currents in a human iPSC-derived neuron before and during TCN 201 (3 μM). Histogram (right) shows the effect of TCN 201 on NMDAR currents in human iPSC-derived neurons. *n* = 5, *P* = 0.012 using paired *t-*test.

Because GluN2 subunit composition is a major factor in determining the physiological characteristics of NMDAR-mediated currents, we investigated the subunit composition of NMDARs in human iPSC-derived neurons. We found that human iPSC-derived neurons expressed both GluN2A- and GluN2B immunofluorescence (Supplementary Fig. S5). But, the decay time (Figs 1e and 3c and Supplementary Table S3) of the NMDAR component of the mEPSCs was in the range for that of NMDARs composed of GluN2B subunits^2^. We therefore tested for the presence of these subunits by using the selective GluN2B antagonist Ro 25-6981^19^. We found that NMDA-evoked currents were dramatically decreased by Ro 25-6981 (1 μM): NMDAR currents were decreased from −807.0 ± 136.1 pA to −238.1 ± 62.1 pA (*n* = 8 cells, *P* < 0.01), which corresponds to an approximately 70% decrease (Fig. 4a). The residual Ro 25-6981-resistant NMDA-evoked currents were dramatically reduced (−238.1 ± 62.1 pA *versus* −13.4 ± 6.3; *n* = 8, *P* < 0.01) by NVP-AAM077 (0.4 μM), an antagonist^20^ of GluN2A-containing NMDARs (Fig. 4a).

To examine GluN2A subunit function of NMDARs by a different approach in human iPSC-derived neurons, we investigated the effect of divalent cation Zn^2+^, selectively blocking GluN2A- but not GluN2B-, 2C, or 2D-containing NMDARs with IC_50_ at nanomolar concentration^21^, and tricine, a chelator of Zn^2+^, on the L-aspartate-evoked currents. The administration of tricine at 10 mM to extracellular recording solutions did not cause any change (−0.38 ± 0.15 nA *versus* −0.38 ± 0.14; *n* = 3, *P* = 0.9) in NMDAR currents (Fig. 4b), suggesting that there was no Zn^2+^ contamination in our recording solutions, which can mask GluN2A subunit-containing NMDAR currents. On the other hand, administering Zn^2+^ extracellularly (free [Zn^2+^] 200 nM) caused a small but significant decrease in L-aspartate-evoked currents from −459.2 ± 96.0 pA to −343.6 ± 70.2 pA (*n* = 5, *P* = 0.014; Fig. 4c), which corresponds to a 25% decrease. Furthermore, we examined the effect of TCN 201, a recently developed, highly selective GluN2A antagonist: at the concentration used for TCN 201 (3 μM) and for glycine (3 μM), TCN 201 has been shown to produce approximately 90% reduction in GluN1/2A currents with no effect on GluN1/2B currents^22^. TCN 201 caused a small inhibitory effect on NMDAR-mediated currents, from −480.2 ± 150.3 pA to −412.5 ± 136.8 pA (*n* = 5, *P* = 0.012; Fig. 4d). Consistently, results from quantitative RT-PCR showed that the expression of *Grin2A* mRNA (which encodes GluN2A subunits) was significantly lower than *Grin2B* mRNA (which encodes GluN2B subunits) (Supplementary Fig. S6).

Together, these findings indicate that NMDAR-mediated currents are dominated by GluN2B subunit-containing receptors in human iPSC-derived neurons and that GluN2A-containing NMDARs play a minor role.

### Protein tyrosine kinase Fyn regulates NMDAR currents in human iPSC-derived neurons

**Figure 5:**
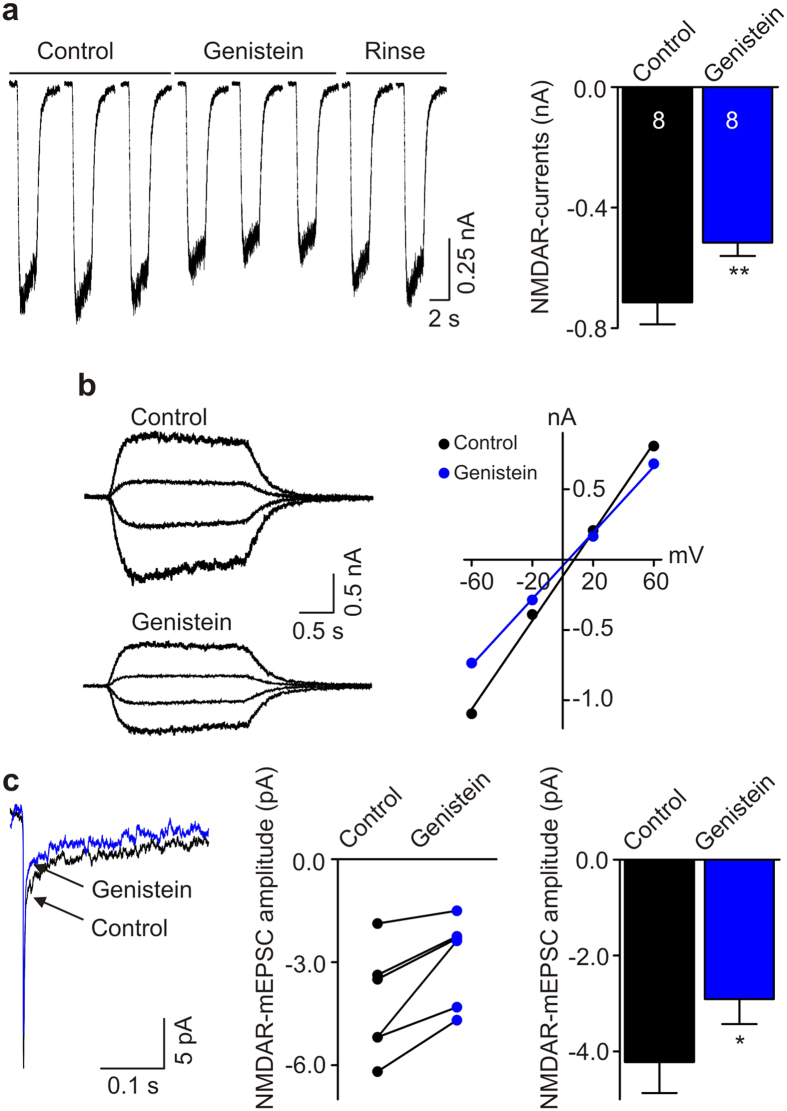
Tyrosine-protein kinase inhibitor genistein suppresses NMDAR currents in human iPSC-derived neurons. (**a**) Left, Representative traces show that genistein reversibly inhibited NMDAR currents in a human iPSC-derived neuron. Right, Histogram showing the effect of genistein on NMDAR currents. *n* = 8, **P = 0.005 using paired *t-*test. (**b**) Left, Typical traces showing NMDAR currents elicited at a series of membrane potentials from −60 mV to +60 mV (in 40 mV increments) before (upper) and after (bottom) adding genistein. Right, A plot shows effect of genistein on current-voltage relationship of NMDAR currents in this human iPSC-derived neuron. (**c**) Left, Averaged mEPSCs recorded at −60 mV before and after adding genistein in a human iPSC-derived neuron. Middle, A graph shows the amplitude of NMDAR-mEPSCs before and after adding genistein in 6 recorded neurons. Right, Histogram showing the effect of genistein on NMDAR-mEPSC amplitude recorded at −60 mV. *n* = 6, *P = 0.012 using paired *t*-test.

NMDARs in rodent neurons are regulated by Src kinases^5^,^23^, a family of non-receptor tyrosine kinases. Therefore, we examined the possibility that tyrosine kinases, and specifically Src family kinases, might regulate NMDAR currents in human iPSC-derived neurons. In order to determine whether NMDARs in these neurons are regulated by tyrosine kinases we used a broad-spectrum tyrosine kinase inhibitor, genistein^24^. We found that genistein (50 μM) reversibly suppressed NMDA-evoked currents: NMDAR currents were decreased from −714.6 ± 72.7 pA to −515.8 ± 44.3 pA (*n* = 8, *P* = 0.005; Fig. 5a). Genistein did not change the reversal potentials of NMDA-evoked currents but rather decreased NMDA-induced conductance (Fig. 5b). Thus, the reduction of NMDA currents by genistein indicates that ongoing tyrosine kinase activity tonically upregulates NMDAR function in human iPSC-derived neurons. We also examined the effect of genistein on the NMDAR component of mEPSCs and found that genistein decreased the amplitude from −4.2 ± 0.6 pA to −2.9 ± 0.5 pA (*n* = 6, *P* = 0.012; Fig. 5c). Hence, we conclude that synaptic NMDARs are tonically upregulated by tyrosine kinase activity.

**Figure 6:**
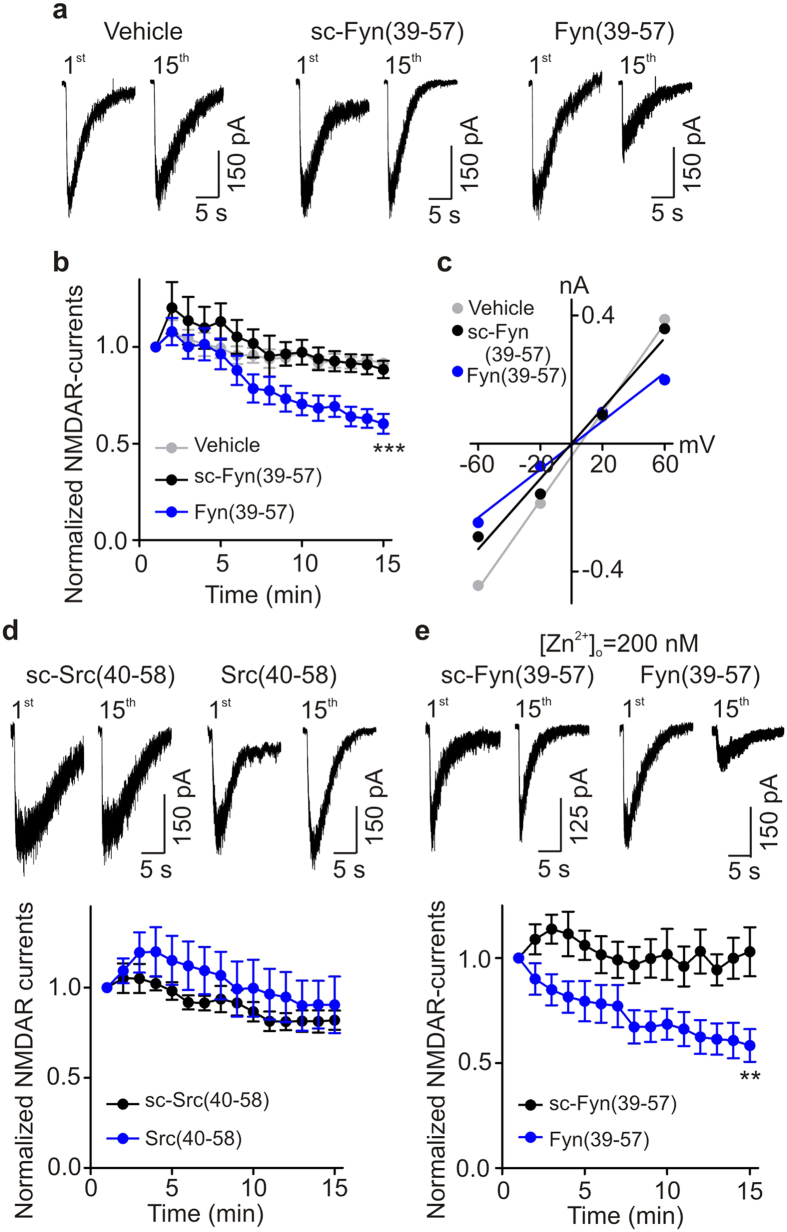
Fyn-interfering peptide Fyn(39–57) inhibits NMDAR currents in human iPSC-derived neurons. (**a**) Representative traces show effect of scrambled Fyn(39–57) (30 μg/ml) and Fyn(39–57) (30 μg/ml) on NMDAR currents in human iPSC-derived neurons. (**b**) A plot showing average NMDAR currents under vehicle alone (*n* = 6), treated with scrambled Fyn(39–57) (*n* = 6), and treated with Fyn(39–57) (*n* = 12). Scrambled Fyn(39–57) and Fyn(39–57) were added *via* the patch pipette. ***P = 0.0005 (using one-way ANOVA). (**c**) A plot shows the effect of scrambled Fyn(39–57) and Fyn(39–57) on the current-voltage relationship of NMDAR currents elicited at a series of membrane potentials from −60 mV to + 60 mV (in 40 mV increments) in human iPSC-derived neurons. (**d**) Representative traces show effect of scrambled Src(40–58) (30 μg/ml) and Src(40–58) (30 μg/ml) on NMDAR currents in human iPSC-derived neurons. A plot showing average NMDAR currents treated with scrambled Src(40–58) (*n* = 6), and treated with Src(40–58) (*n* = 9). *P* > 0.05. (**e**) Typical traces showing effect of scrambled Fyn(39–57) and Fyn(39–57) on NMDAR currents in human iPSC-derived neurons in the presence of 200 nM free Zn^2+^. A plot shows average NMDAR currents treated with scrambled Fyn(39–57), and treated with Fyn(39–57) in the presence of 200 nM free Zn^2+^. *n* = 8 each, **P = 0.007.

Src kinase and Fyn kinase, two non-receptor tyrosine-protein kinases of Src family, are known regulators of NMDARs^23^. We therefore investigated whether either of these kinases contributes to the tyrosine-kinase mediated upregulation of NMDARs in human iPSC-derived neurons. To test for Fyn regulation, we included Fyn(39–57), a Fyn-interfering peptide^25^, in the patch recording electrode pipette. We found that in neurons treated with Fyn(39–57) L-aspartate-evoked currents progressively decreased to 60.2 ± 5.1% of the initial level by 15 minutes after the first response (Fig. 6a,b). Fyn(39–57) did not, however, change the reversal potential of NMDAR currents (Fig. 6c). In contrast to neurons treated with Fyn(39–57), in neurons recorded with scrambled Fyn(39–57) NMDAR currents 15 minutes after the first response were 88.4 ± 4.5% of the initial level (*P* < 0.01 compared with Fyn(37–59)) (Fig. 6b). In addition, normalized NMDAR currents 15 minutes after the first response in neurons treated with intracellular vehicle alone were significantly greater (91.6 ± 1.5% *versus* 60.2 ± 5.1%; *P* < 0.01) than those in neurons treated with Fyn(39–57) (Fig. 6a,b; *P* < 0.01). From these findings we conclude that Fyn kinase contributes to the ongoing upregulation of NMDAR-mediated currents.

Next, we investigated the possible involvement of Src kinase in the upregulation of NMDARs by including Src(40–58), a Src-interfering peptide^6^, in the recording pipette. We found that Src(40–58) did not have any effect on NMDAR currents in human iPSC-derived neurons: normalized L-aspartate-evoked currents 15 minutes after the first response to the agonist were 81.9 ± 5.3% and 90.4 ± 1.6% (*P* > 0.05) after treatment by scrambled Src(40–58) or by Src(40–58), respectively (Fig. 6d). Furthermore, we tested whether Src kinase might regulate GluN2A-mediated currents in human iPSC-derived neurons. We isolated GluN2A-mediated currents by blocking GluN2B-containing receptors (recordings in the continuous presence of Ro 25-6981 at 1 μM) and then examined the effect of Src(40–58). We found that Src(40–58) did not have any effect on L-aspartate-evoked currents in the presence of Ro 25-6981: normalized NMDAR currents 15 minutes after the first response to the agonist were 98.3 ± 8.7% (*n* = 3, *P* > 0.05) after treatment by Src(40–58) (Supplementary Fig. S7).

Thus, our results show that there is ongoing regulation of NMDARs by Fyn, but not Src, kinase in human iPSC-derived neurons. In hippocampal neurons from rats, Fyn kinase regulates GluN2B subunit-containing NMDARs whereas Src kinase regulates GluN2A subunit-containing NMDARs^25^. To determine whether Fyn regulates GluN2B-containing or GluN2A-containing NMDARs in human iPSC-derived neurons, we blocked GluN2A subunit-containing NMDARs by adding free Zn^2+^ (200 nM) to the extracellular solutions. We found that in the presence of Zn^2+^, 15 minutes after the first response NMDAR currents were 58.4 ± 7.8% of the initial level in cells treated with Fyn(39–57), but NMDAR currents remained at 103.0 ± 11.6% in cells treated with scrambled Fyn(39–57) (Fig. 6e). Thus, Fyn(39–57) still suppressed NMDAR-mediated currents in human iPSC-derived neurons when GluN2A-containing receptors were blocked. We conclude therefore that the ongoing upregulation by Fyn kinase is through GluN2B-containing NMDARs in human iPSC-derived neurons.

## Discussion

We investigated the properties of human NMDARs *in situ* in iPSC-derived neurons. Our results indicate that human iPSC-derived neurons express GluN2B subunit-dominant NMDARs, the function of which is regulated by tyrosine-protein kinase Fyn. This is the first evidence demonstrating that Fyn kinase regulates function of NMDARs in human iPSC-derived neurons.

Our conclusion that the NMDARs expressed in human iPSC-derived neurons are dominated by receptors containing GluN2B subunits is based on three lines of evidence following from the known pharmacological and biophysical differences in NMDARs with differing GluN2 subunit composition^2^. First, we found that NMDAR-mediated currents in human iPSC-derived neurons were inhibited by approximately 70% by the GluN2B-selective blocker Ro 25-6981 which we used at a concentration known to inhibit GluN2B-containing receptors without affecting GluN2A, 2C, or 2D NMDARs^19^. The remainder of the currents were blocked by NVP-AAM077 which preferentially suppresses GluN2A-containing receptors^20^. Moreover, extracellular administration of Zn^2+^, which also differentially suppresses GluN2A-containing receptors^2^, blocked only 25% of the NMDA currents. In addition, TCN 201 reduced NMDAR-evoked currents by only 16%. Taking the small effects of NVP-AAM077, Zn^2+^ and TCN 201 together with the large effect of Ro 25-6981 provides pharmacological evidence that GluN2B is the dominant subtype of NMDAR.

Second, the results from quantitative PCR indicate that human iPSC-derived neurons express significantly higher level of *Grin2B* mRNA than *Grin2A* mRNA. Our third line of evidence concerning GluN2 subunit composition relates to the decay time constant of the NMDAR-mediated component of the mEPSCs. GluN2A- and GluN2B-subunit-mediated NMDARs can be distinguished from each other by the decay time constant of the receptor-mediated synaptic currents. GluN2B-NMDAR-mediated synaptic currents in the CNS have decay time constants in the hundreds of milliseconds, whereas GluN2A-NMDAR-mediated synaptic currents have decay time constants below 100 milliseconds^26^. The decay time constant of the NMDAR component of mEPSCs we observed in human iPSC-derived neurons was in the hundreds of milliseconds (Figs 1e and 3c and Supplementary Table S3), indicating that these synaptic NMDAR-mediated currents are dominated by receptors containing GluN2B subunits. Collectively, the decay time constant observations together with our findings from pharmacology and quantitative PCR represent a coherent body of evidence that GluN2B is the dominant subtype of NMDAR and that GluN2A-containing NMDARs play a minor role in human iPSC-derived neurons.

Expression of GluN2 subunits, including two predominant subunits, GluN2A and GluN2B, is spatiotemporally controlled in the CNS. A striking feature is the occurrence of a switch from GluN2B subunits to GluN2A subunits in the CNS during neuronal development^27^,^28^: a dramatic increase in GluN2A subunits accompanying a decrease in GluN2B subunits especially in synapses. In most regions in the mature CNS, GluN2B-containing NMDARs are considered to be localized principally at extrasynaptic sites^29^. However, in some CNS regions GluN2B subunits dominate synaptic responses at mature synapses^30^. Our findings that GluN2B subunit-containing NMDARs are dominant in human iPSC-derived neurons may be attributed to these neurons being in a relatively immature state^31^. Alternatively, the GluN2B dominant phenotype of the neurons may indicate that the neurons reflect specific subpopulations or functional states of neurons in the mature CNS.

GluN2A- and GluN2B-containing NMDARs may have distinct functional roles in the CNS. For example, although controversial, it has been suggested that GluN2A subunit-containing NMDARs mediate the induction of long-term potentiation in the CA1 region of the hippocampus, whereas GluN2B subunit-containing NMDARs mediate the induction of long-term depression^32^,^33^,^34^. It may be that the differential role of the receptor subtypes is a form of metaplasticity^35^ that depends on distinct regulation of GluN2A subunit- and GluN2B subunit-containing NMDARs by different Src family kinases^25^: Src kinase upregulation of the activity of GluN2A subunit-containing NMDARs is necessary for potentiation, but Fyn kinase upregulation of activity of GluN2B subunit-containing NMDARs is required for depression. In our present study we find that there is cross-species conservation not only of upregulation of NMDARs by Src family kinases but also of the subunit-kinase coupling of Fyn upregulation to GluN2B-containing NMDARs in human iPSC-derived neurons. Thus, a core molecular process for synaptic metaplasticity that has been identified in rodent neurons^25^–regulation of NMDARs by tyrosine phosphorylation–may occur in human neurons.

Our findings indicate that there is ongoing activation of Fyn kinase in human iPSC-derived neurons. The upregulation of NMDAR currents by Fyn kinase might be mediated directly, that is through direct phosphorylation of consensus phosphorylation sites on GluN2B^36^,^37^, but we cannot exclude indirect effects of Fyn through phosphorylation of other proteins which themselves regulate NMDAR activity. This ongoing activation may be due to neurotrophic factors that are included in the neuronal culture medium. For example, brain-derived neurotrophic factor (BDNF) has been reported to increase channel activity of NMDARs containing GluN2B subunits through Fyn-mediated phosphorylation at tyrosine 1472 of GluN2B subunits^36^,^37^. However, the possibility remains that other factors during differentiation of human iPSC-derived neurons cause the activation of Fyn kinase.

Our results have important implications for the use of iPSC-derived neurons in disease modeling and drug screening. NMDAR dysfunction is thought to underlie several disorders of the brain^2^. Our findings suggest that iPSC-derived neurons are suitable for modeling disorders that result from misregulation of GluN2B, such as ischemia and Alzheimer’s disease^2^. Mutations of *GRIN2B* have been reported in several studies of autism spectrum disorder (ASD)^38^,^39^, but the functional consequences of these mutations are unknown. Our data suggest that human iPSC-derived neurons may be particularly useful for exploring the roles of these mutations and other mutations that alter NMDAR signaling^40^ in the development of ASD. iPSC-derived neurons also have tremendous potential for their use in drug screening. NMDARs are attractive candidate targets for drug development, and several peptides and small molecules that modulate NMDAR signaling are currently under investigation in clinical and pre-clinical trials for a variety of disorders^2^. Our data suggest that human iPSC-derived neurons may be useful for identification of novel modulators of GluN2B *in situ* using human neurons.

In summary, our studies show that NMDAR-mediated currents are dominated by receptors containing GluN2B subunits and that activation of Fyn kinase potentiates the currents in human iPSC-derived neurons. This work supports efficiency of human iPSC-derived neuron-based model system and our findings also provide a basis for utilizing human iPSC-derived neurons in screening for drugs targeting NMDARs in neurological disorders.

## Materials and Methods

### Generation of human iPSCs and neuronal differentiation

Donated fibroblasts were obtained with informed consent for reprogramming approved by the SickKids Research Ethics Board and the Canadian Institutes of Health Research Stem Cell Oversight Committee. *In vivo* teratoma experiments were approved by the SickKids Animal Care Committee. All studies were performed under the regulation of the SickKids Research Ethics Board and Canadian Institutes of Health Research Stem Cell Oversight Committee. Human iPS cells were generated as described^17^. Human iPSC characterization was performed as previously described^11^,^41^. Human iPSCs were directed to differentiate into neurons using our previously published “Brennand” protocol^11^, which was adapted from another published protocol^10^. Unless otherwise indicated all cell culture reagents were acquired from Thermo Scientific. In brief, iPSCs were grown as embryoid bodies for one week in the presence of SB431542 (10 μm, Stemgent) and dorsomorphin (2 μM, Sigma) to promote ectoderm specification. Embryoid bodies were then seeded on plates coated with poly-L-ornithine (Sigma, 0.1mg/ml) and laminin (Roche, 10 μg/ml) to promote the formation of neural rosettes; all subsequent steps employed plates coated with poly-L-ornithine and laminin. One week later, rosettes were manually dissected and then dissociated with Accutase (Innovative Cell Technologies) to release neural precursor cells (NPCs). NPCs were grown for several passages in media composed of: DMEM-F12 supplemented with N2 and B27, non-essential amino acids, heparin (2 μg/ml, Sigma), FGF2 (10 ng/ml, R&D), 1 μg/ml laminin and penicillin/streptomycin. To promote neuronal differentiation, NPCs were seeded at low density (50,000 cells per 2 cm^2^) in complete neuronal differentiation medium: Neurobasal medium supplemented with N2 and B27, non-essential amino acids, laminin, penicillin /streptomycin mixtures (GIBCO), BDNF (10 ng/ml, Peprotech Inc), GDNF (10 ng/ml, Peprotech Inc), IGF-1 (10 ng/ml, Peprotech Inc), cAMP (1 μM, Sigma), and ascorbic acid (200 ng/ml, Sigma). The cells were placed in the incubator at 37 °C and the culture medium was changed every two days.

### Electrophysiology

Whole-cell patch-clamp recordings were performed at room temperature (22 °C) in human iPSC-derived neurons (7–11 weeks in culture). An Axopatch 1-D amplifier (Molecular Devices, USA) and a DigiData 1200 series interface (Molecular Devices, USA) were used. Electrical signals were digitized at 10 kHz and filtered at 2 kHz. Recording electrodes were pulled from a P-87 pipette puller (Sutter Instrument Co., USA), using micropipettes (World Precision Instruments, Inc., USA), and had resistances of 5 to 8 MΩ. Intracellular recording solutions, for the recordings on membrane properties and spontaneous synaptic activities, were composed of (in mM): 144 K^+^-gluconate, 10 KCl, 10 HEPES, 2 EGTA, and 2 Mg-ATP, and pH was adjusted to 7.20 with KOH. The extracellular recording solutions were composed of (in mM): 140 NaCl, 5.4 KCl, 2 CaCl_2_, 1 MgCl_2_, 15 HEPES, and 10 glucose, and pH was adjusted to 7.35 with NaOH. A liquid junction potential of 16 mV was subtracted from the membrane potential values measured with K^+^-gluconate based internal solutions under current-clamp condition. The membrane potentials under voltage-clamp condition were held at −70 mV and voltage-gated currents were evoked by stepping the membrane potentials to a series of potentials from −80 mV to +60 mV (in 10 mV increments) for 400 ms. Action potentials under current-clamp condition with membrane potentials of around −75 mV were evoked by injection of a series of current steps from −5 pA to +50 pA (in 5 pA increments) for 1 s. For the recordings of GABA_A_ receptor- and glycine receptor-mediated currents, 0.5 μM TTX and 10 μM SCH 50911 were added to the extracellular recording solutions. GABA_A_ receptor and glycine receptor-mediated currents were elicited, under voltage-clamp condition with −70 mV of holding potentials, by focal puffing of 0.5 mM GABA (Sigma, USA) and 0.5 mM glycine (Sigma, USA) using a Picospritzer II (General Valve Corporation, USA) for 100 ms, respectively.

Glutamate receptor- and NMDAR-mediated currents were recorded at membrane potentials of −60 mV under voltage-clamp condition. The extracellular solutions were composed of (in mM): 140 NaCl, 5.4 KCl, 15 HEPES, 25 glucose, 0.0005 tetrodotoxin, 1.3 CaCl_2_, 0.003 glycine, and pH was adjusted to 7.35 with NaOH. The intracellular solutions contained (in mM): 120 CsF, 18.5 CsCl, 10 HEPES, 10 BAPTA, 4 Mg-ATP, and pH was adjusted to 7.20 with CsOH. Glutamate receptor-mediated currents were elicited by focal puffing of 0.5 mM glutamate (Sigma, USA) for 100 ms. NMDAR-mediated currents were elicited by focal puffing of 0.25 mM L-aspartic acid sodium salt (Sigma, USA; dissolved in extracellular solution) for 50 ms or by 50 μM NMDA (Sigma, USA) using fast-step perfusion system (SF-77B, Warner Instruments, USA). Free Zn^2+^ (200 nM) was obtained by using tricine (10 mM) to buffer zinc following the formula: [Zn^2+^]_free_ = [Zn^2+^]_added_/200^21^. Genistein was continuously applied through the barrel with control recording solution, but was not in the solution containing NMDA of fast-step perfusion system. For the recordings of mEPSCs, 10 μM bicuculline and 1 μM strychnine were added to the extracellular recording solutions above. mEPSCs were detected and analyzed using Mini Analysis Program (Synaptosoft Inc, NJ, USA). The amplitude of mEPSCs was measured by the peak current 15 ms after the AMPAR-mediated peak value^7^.

Prism sheets (GraphPad software, CA, USA) were used and data were shown as mean ± SEM. Statistical analysis was performed by using Student’s paired or unpaired *t*-test, or one-way ANOVA, as appropriate, and the difference was regarded to be significant when *P* value was less than 0.05.

## Additional Information

**How to cite this article**: Zhang, W.-B. *et al.* Fyn Kinase regulates GluN2B subunit-dominant NMDA receptors in human induced pluripotent stem cell-derived neurons. *Sci. Rep.* **6**, 23837; doi: 10.1038/srep23837 (2016).

## Supplementary Material

Supplementary Information
