# Supplementary material for: Fyn Kinase regulates GluN2B subunit-dominant NMDA receptors in human induced pluripotent stem cell-derived neurons
Source: Sci Rep. 2016 Apr 4;6:23837. doi: 10.1038/srep23837 (PMC4819183; doi:10.1038/srep23837)
Supplement: Supplementary Information [file srep23837-s1.pdf]

Kinase Fyn regulates GluN2B subunit-dominant NMDA receptors in human induced pluripotent stem cell-derived neurons

Wen-Bo Zhang[1,2], P. Joel Ross[3], YuShan Tu[1], Yongqian Wang[1], Simon Beggs[1,2], Ameet S. Sengar[1], James Ellis[3,4], Michael W. Salter[1,2*]

[1]Program in Neurosciences & Mental Health, The Hospital for Sick Children, Toronto, ON, M5G 0A4, Canada

[2]Department of Physiology, University of Toronto, Toronto, ON, Canada

[3]Program in Developmental & Stem Cell Biology, The Hospital for Sick Children, Toronto, ON, M5G 0A4, Canada.

[4]Department of Molecular Genetics, University of Toronto, Toronto, ON, Canada.

* Corresponding author: mike.salter@utoronto.ca

Phone: 416-813-6272
Fax: 416-813-5085

Immunocytochemistry

Human iPSC-derived neurons were fixed with 4% PFA, permeabilized with 0.1% Triton for 5 min and blocked with 10% normal donkey serum for 1 h. The neurons were then incubated overnight with primary antibodies against GluN2A (1:1000, BD Biosciences), GluN2B (1:500, BD Biosciences) and MAP2 (1:3000, BioLegend), followed by incubation with appropriate fluorescently labelled secondary antibodies (1:3000, Jackson ImmunoResearch Labs) for 2 h. Sections were mounted with DAPI-containing medium (Sigma) and imaged using a Quorum OptiGrid Epifluorescence microscope (Quorum Technologies) and Perkin Elmer Volocity software.

Real-Time PCR analysis

Total RNA was purified from cultured human iPSC-derived neurons using the PureLink® RNA Mini Kit™ (Thermo Scientific, USA). cDNA was synthesized from total RNA using the Maxima™ First Strand cDNA Synthesis Kit (Thermo Scientific, USA). Real-time PCR primers were designed around exon-intron junctions or large introns to prevent amplification of genomic DNA. Primer sequences are listed in Supplementary Table S2. Real-time PCR was performed using the KAPA™ SYBR Fast qPCR Kit (KAPA Biosystems, USA) and StepOnePlus™ Real-Time PCR System (Applied Biosystems, USA). *TBP* and *SDHA* were used as endogenous controls to quantify gene expression levels. The ΔCt values were determined by StepOne™ and StepOnePlus™ Software v2.3 (Applied Biosystems, USA). Relative ratios for each mRNA were normalized by *TBP* and *SDHA*. The student *t*-test was used to compare the relative ratios for each mRNA.

Supplementary Figure S1

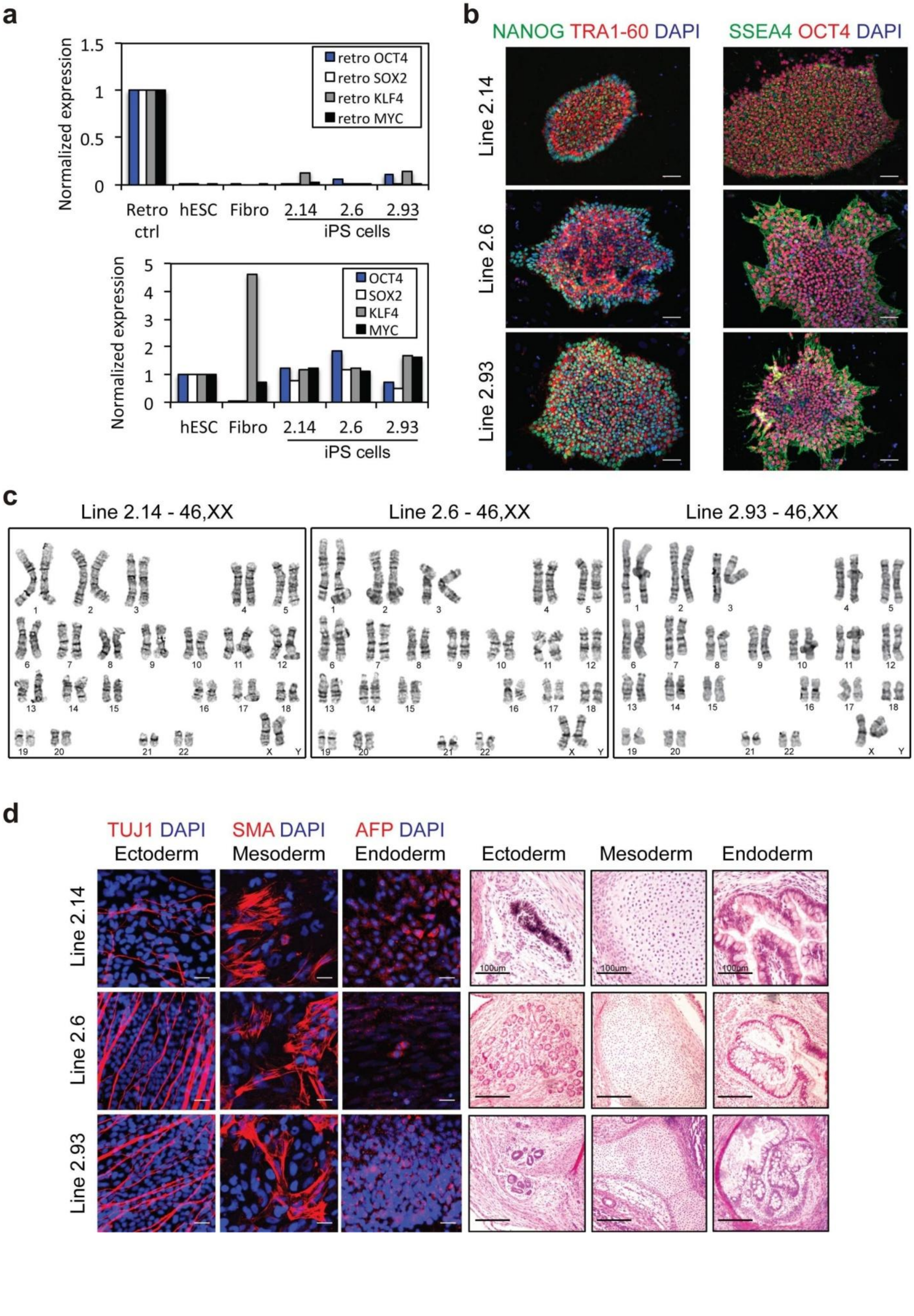

Supplementary Figure S1. Characterization of novel iPS cell lines from a healthy adult female. (**a**) iPSC lines repress the viral reprogramming factors and induce expression of endogenous pluripotency genes. Gene expression was analyzed by qRT-PCR with the indicated primer sets and data (from a single experiment) were normalized to human fibroblasts that were infected with the viral reprogramming factors (top) or human embryonic stem cells (hESC; bottom). (**b**) iPSCs synthesize pluripotency-associated proteins. iPSCs were fixed and labeled by immunocytochemistry with the indicated antibodies; the scale bar represents 50 µm. (**c**) iPSCs have normal karyotypes. G-banded metaphase chromosomes are displayed. (**d**) iPSCs are functionally pluripotent. iPSCs lines are pluripotent in vitro (embryoid body assay, left) and in vivo (teratoma assay, right). Embryoid bodies were labeled by immunocytochemistry with the indicated markers of the three germ layers; scale bar represents 20 µm (left). Sectioned teratomas revealed the formation of three dimensional structures that are typical of the three germ layers; scale bar represents 100 µm (right).

Supplementary Figure S2

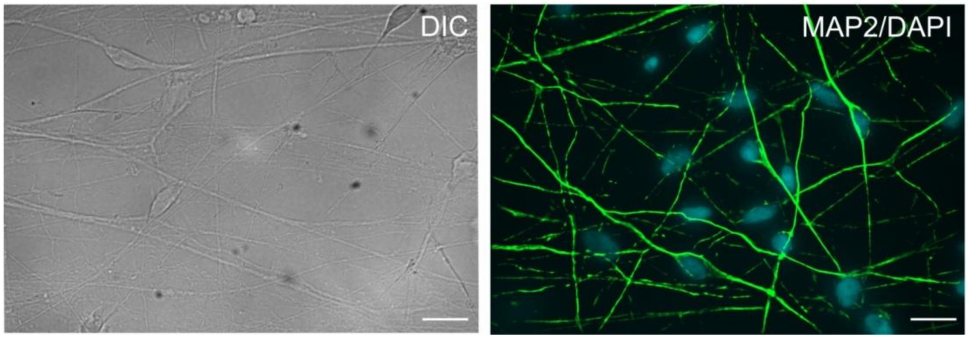

Supplementary Figure S2. Human iPSC-derived neurons have neuronal morphology and express neuron-specific protein MAP2. A DIC image (left) and an immunofluorescence image (right) showing immunoreactivity to anti-MAP2 (green) and DAPI (blue) nuclear stain. Scale bar: 10 µm.

Supplementary Figure S3

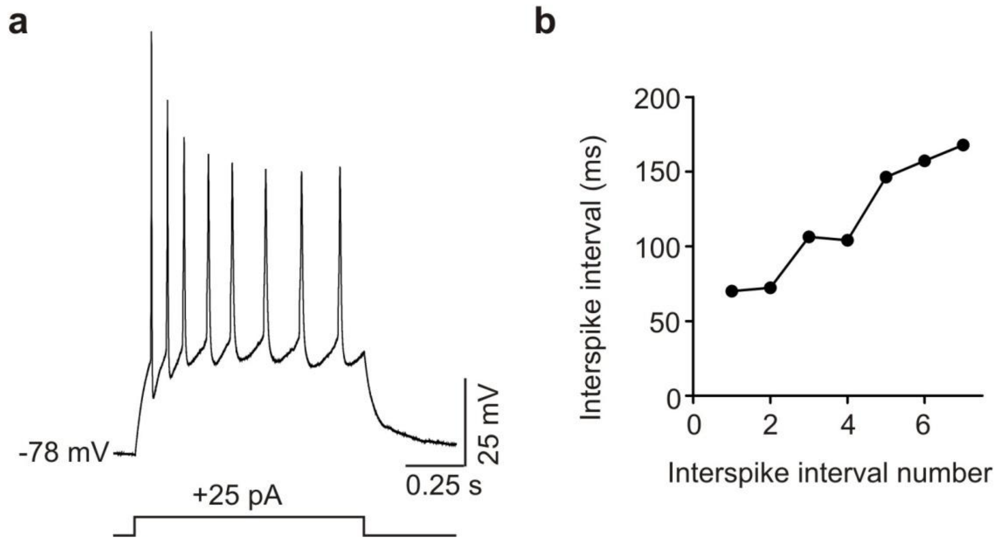

Supplementary Figure S3. Evoked action potentials display spike frequency adaptation in human iPSC-derived neurons. (**a**) Typical traces display action potentials evoked by injecting a current step of +25 pA for 1 s in a human iPSC-derived neuron. (**b**) A plot shows the relationship of interspike interval and interspike interval number in a neuron.

Supplementary Figure S4

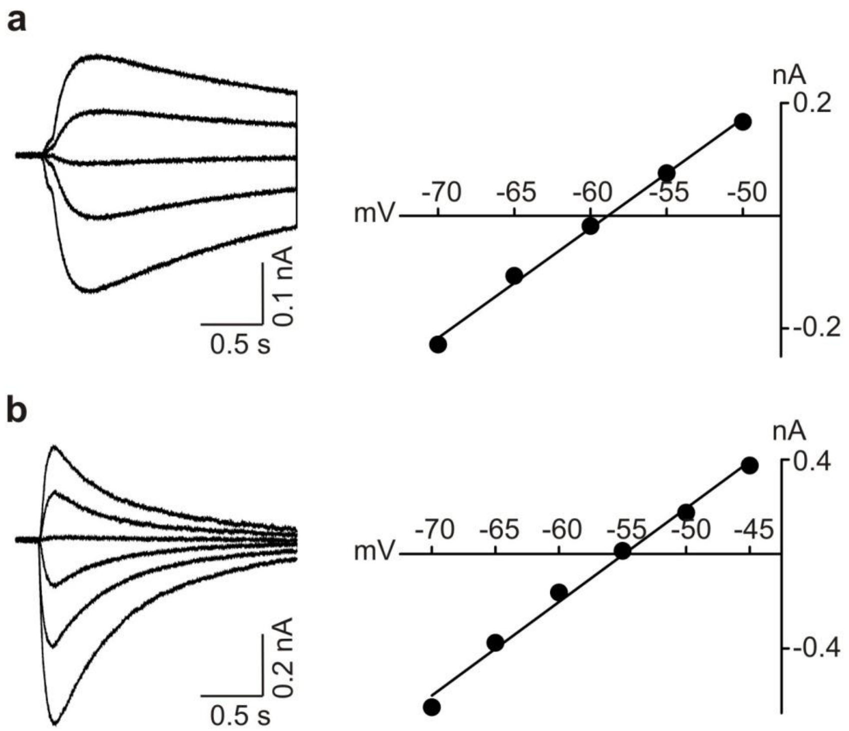

Supplementary Figure S4. The reversal potentials of ligand-gated receptors. (**a**) The reversal potentials of GABA-evoked currents in a human iPSC-derived neuron. (**b**) The reversal potentials of glycine-evoked currents in a human iPSC-derived neuron.

Supplementary Figure S5

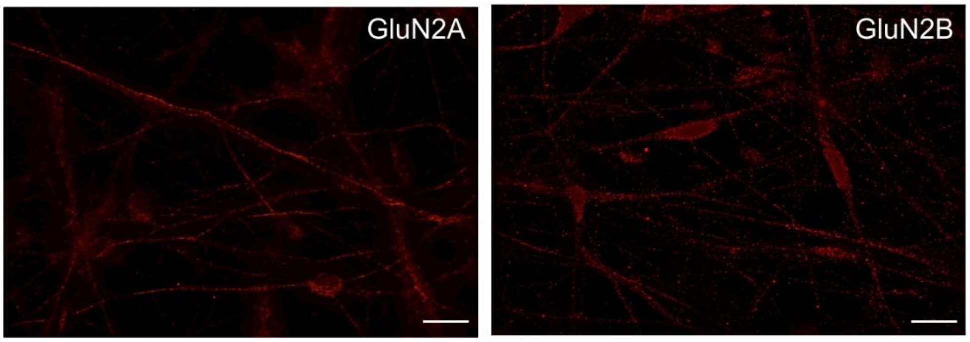

Supplementary Figure S5. Human iPSC-derived neurons express GluN2A and GluN2B subunits. Immunofluorescence images show immunoreactivity to anti-GluN2A (left) and anti-GluN2B (right) in human iPSC-derived neurons. Scale bar: 10 µm.

Supplementary Figure S6

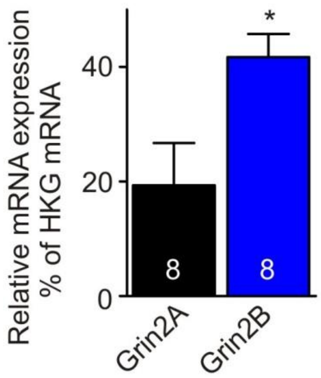

Supplementary Figure S6. Relative mRNA expression of *Grin2A* and *Grin2B* in human iPSC-derived neurons. Histogram shows the relative mRNA expression of *Grin2A* and *Grin2B* (% of mRNA of Housekeeping genes, *TBP* and *SDHA*). *n*=8 each, **P* = 0.02.

Supplementary Figure S7

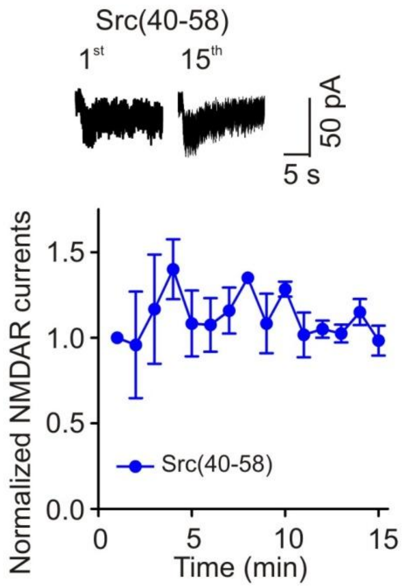

Supplementary Figure S7. Src-interfering peptide Src(40-58) does not have any effects on NMDAR currents. Upper, Representative traces show effect of Src(40-58) on NMDAR currents recorded in the continuous presence of Ro 25-6981 (1 µM) in a human iPSC-derived neuron. Bottom, A plot showing average NMDAR currents recorded under treated with Src(40-58) in the continuous presence of Ro 25-6981 (1 µM). (*n*=3).

Supplementary Table S1

RMP	-49.2 ± 3.1 mV
Capacitance	42.8 ± 5.4 pF
Input resistance	1.9 ± 0.4 GΩ
Peak Na^+ currents	-1.3 ± 0.2 nA
Peak K^+ currents	1.9 ± 0.2 nA
AP threshold	-45.5 ± 2.8 mV
AP amplitude	77.5 ± 5.6 mV
AP rise time	1.7 ± 0.3 ms
AP half-duration	2.4 ± 0.3 ms
AP decay time	2.1 ± 0.4 ms

Supplementary Table S1. Intrinsic membrane properties in human iPSC-derived neurons.

n=10.

Supplementary Table S2

Gene	Accession	Forward primer	Reverse Primer
Grin2A	NM_000833	aaccatctcagcatcgtcac	tcacattcatccccctcattgg
Grin2B	NM_000834	agagaatctaccagtccaatatgc	ctttcccacttcctctccttg
TBP	NM_003194	gagagttctgggattgtaccg	atcctcatgattaccgcagc
SDHA	NM_004168	tggttgtctttggtcggg	gcgtttggtttaattggaggg

Supplemental Table S2. Primers used for real-time PCR analysis.

Supplementary Table S3

mEPSC amplitude	mEPSC frequency	NMDAR-mEPSC decay time (τ)
-16.6 ± 2.5 pA	0.5 ± 0.1 Hz	278.4 ± 35.7 ms

Supplementary Table S3. Amplitude and frequency of mEPSCs and decay time of NMDAR-mEPSC in human iPSC-derived neurons. *n*=7.
